# Supplementary material for: Dual Cannabinoid and Orexin Regulation of Anhedonic Behaviour Caused by Prolonged Restraint Stress
Source: Brain Sci. 2023 Feb 13;13(2):314. doi: 10.3390/brainsci13020314 (PMC9954020; doi:10.3390/brainsci13020314)
Supplement: Supplementary file 1 [file brainsci-13-00314-s001.zip › brainsci-2159093-supplementary.pdf]

Dual cannabinoid and orexin regulation of anhedonic behaviour caused by prolonged restraint stress

Hye Ji J. Kim [1], Ayat Zagzoog [a], Costanza Ceni [1,2,3], Rebecca Ferrisi [1,2], Nicola Janz [1], Robert B. Laprairie [1,4,*]

[1] College of Pharmacy and Nutrition, University of Saskatchewan, Saskatoon SK, Canada

[2] Department of Pharmacy, University of Pisa, Pisa, Italy

[3] Doctoral School in Life Sciences, University of Siena, Siena, Italy

[4] Department of Pharmacology, College of Medicine, Dalhousie University, Halifax NS, Canada

* Correspondence: robert.laprairie@usask.ca

Supplementary Materials

Supplementary Figures

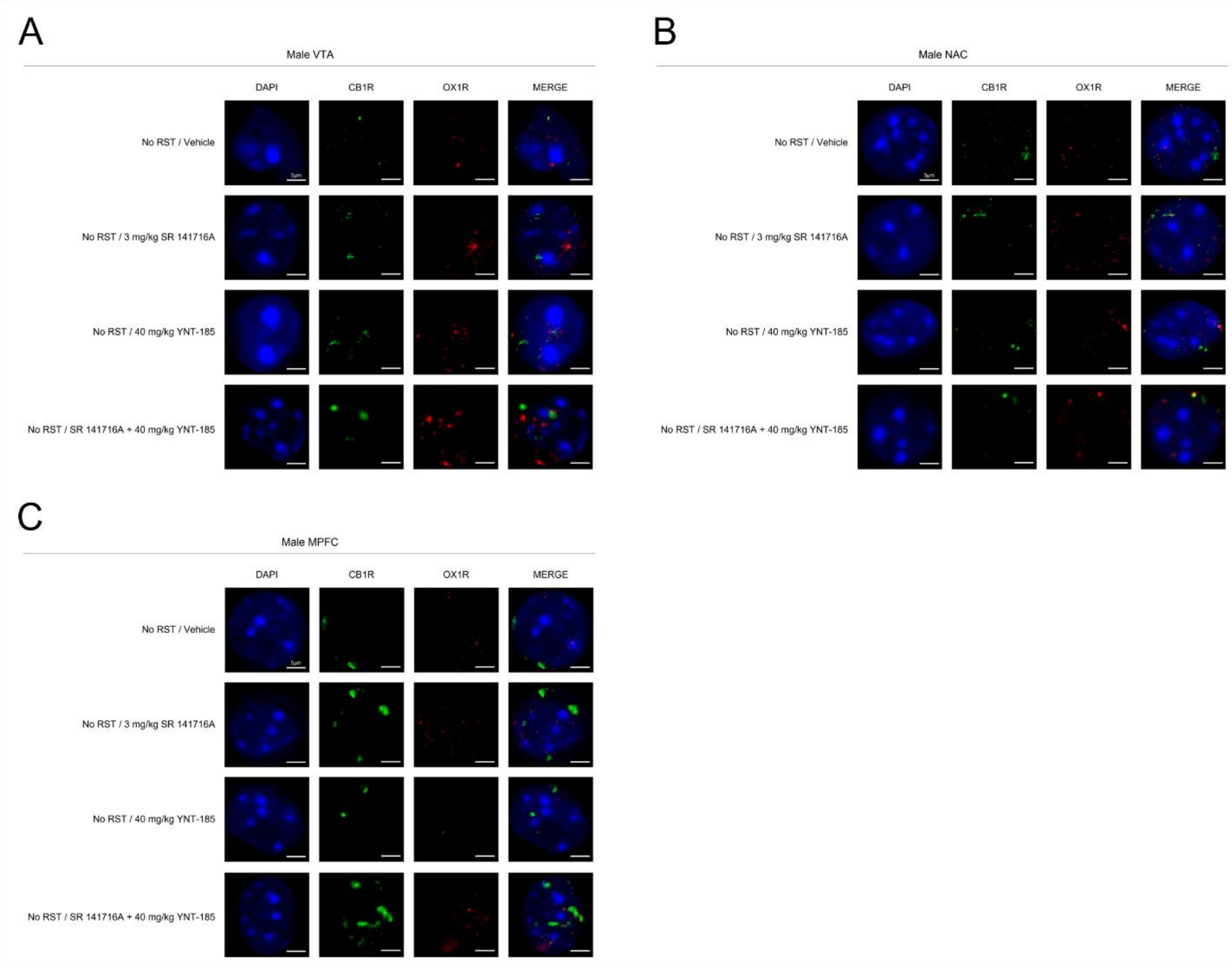

Figure S1. Representative images of CB1R-OX1R colocalization in males. VTA (**A**), NAC (**B**), and MPFC (**C**) tissue. Images were taken on a Zeiss LSM880 confocal microscope then processed using ImageJ (version 2.1.0) with the FIJI extension.

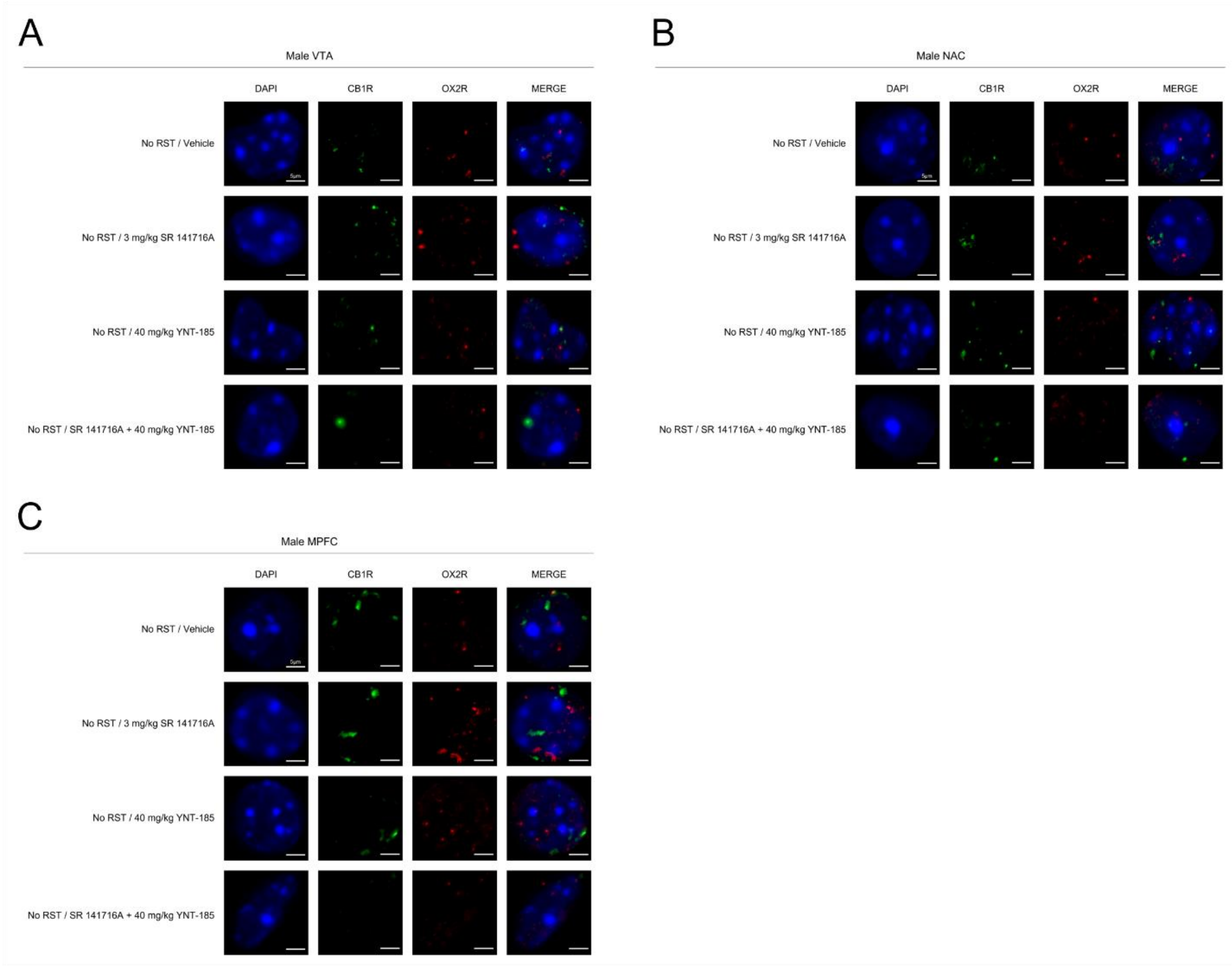

Figure S2. Representative images of CB1R-OX2R colocalization in males. VTA (**A**), NAC (**B**), and MPFC (**C**) tissue. Images were taken on a Zeiss LSM880 confocal microscope then processed using ImageJ (version 2.1.0) with the FIJI extension.

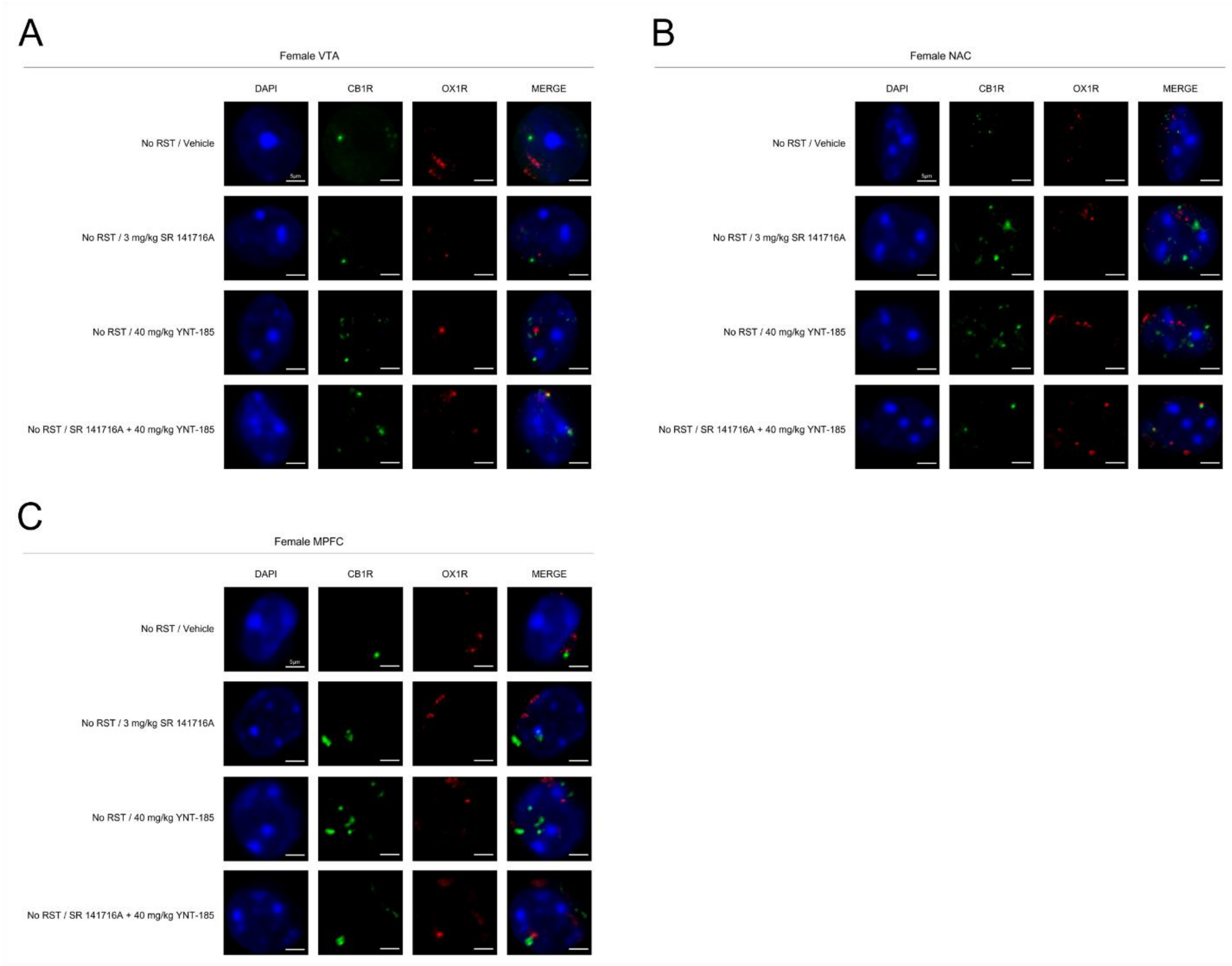

Figure S3. Representative images of CB1R-OX1R colocalization in females. VTA (**A**), NAC (**B**), and MPFC (**C**) tissue. Images were taken on a Zeiss LSM880 confocal microscope then processed using ImageJ (version 2.1.0) with the FIJI extension.

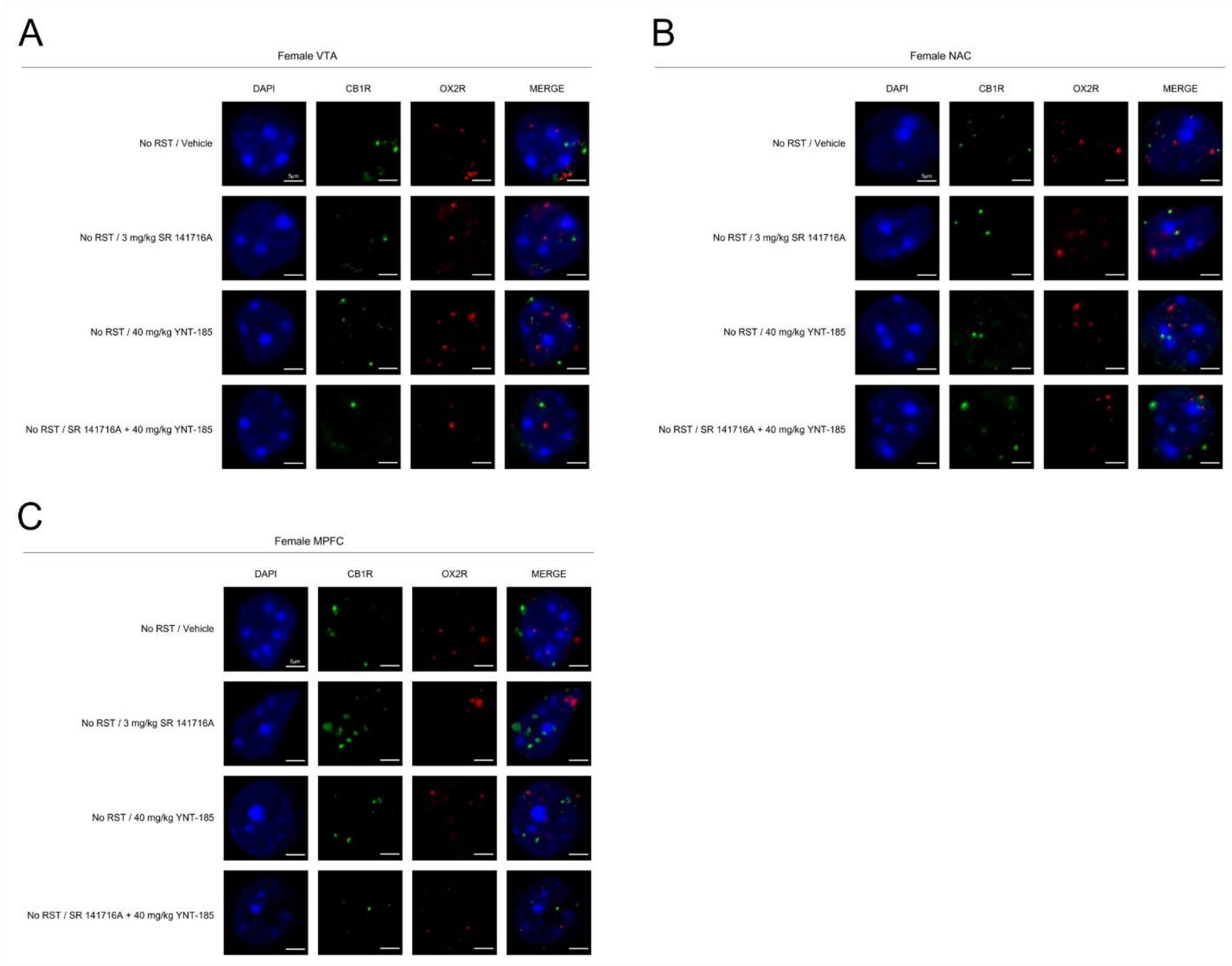

Figure S4. Representative images of CB1R-OX2R colocalization in females. VTA (**A**), NAC (**B**), and MPFC (**C**) tissue. Images were taken on a Zeiss LSM880 confocal microscope then processed using ImageJ (version 2.1.0) with the FIJI extension.

Supplementary Tables

**Table S1.** Figure 2A Multiple Comparisons (p-value)	**RST**	**To Vehicle within -RST**	**To Vehicle within +RST**
Vehicle	<0.001		
0.3 mg/kg CP55,940	<0.001	>0.9999	0.3108
1 mg/kg TCS-1102	<0.001	>0.9999	0.9561
0.3 mg/kg CP55,940 +1 mg/kg TCS-1102	<0.001	>0.9999	0.9779
3 mg/kg SR141716A	0.001	0.9128	0.0048
40 mg/kg YNT-185	0.7458	>0.9999	<0.0001
3 mg/kg SR141716A + 40 mg/kg YNT-185	>0.9999	0.9878	<0.0001

**Table S2.** Figure 2A ANOVA	**F(DFn,DFd)**
RST	F (1, 70) = 197.1
Treatment	F (6, 70) = 9.781
Interaction	F (6, 70) = 10.40

**Table S3.** Figure 2B Multiple Comparisons (p-value)	**RST**	**To Vehicle within -RST**	**To Vehicle within +RST**
Vehicle	<0.0001		
0.3 mg/kg CP55,940	0.0010	>0.9999	>0.9999
1 mg/kg TCS-1102	0.0892	0.7169	>0.9999
0.3 mg/kg CP55,940 +1 mg/kg TCS-1102	0.1901	0.4467	>0.9999
3 mg/kg SR141716A	<0.0001	0.9794	>0.9999
40 mg/kg YNT-185	0.2867	0.9776	0.9909
3 mg/kg SR141716A + 40 mg/kg YNT-185	>0.9999	0.1095	0.3060

**Table S4.** Figure 2B ANOVA	**F(DFn,DFd)**
RST	F (1, 70) = 88.91
Treatment	F (6, 70) = 1.957
Interaction	F (6, 70) = 5.003

**Table S5.** Figure 2C Multiple Comparisons (p-value)	**RST**	**To Vehicle within -RST**	**To Vehicle within +RST**
Vehicle	0.5960		
0.3 mg/kg CP55,940	0.9928	0.8103	0.9997
1 mg/kg TCS-1102	0.9999	0.7269	>0.9999
0.3 mg/kg CP55,940 +1 mg/kg TCS-1102	0.9952	0.8383	>0.9999
3 mg/kg SR141716A	0.9977	0.9336	>0.9999
40 mg/kg YNT-185	>0.9999	0.8482	>0.9999
3 mg/kg SR141716A + 40 mg/kg YNT-185	>0.9999	>0.9999	0.9717

**Table S6.** Figure 2C ANOVA	**F(DFn,DFd)**
RST	F (1, 70) = 7.797
Treatment	F (6, 70) = 2.008
Interaction	F (6, 70) = 0.4506

**Table S7.** Figure 2D Multiple Comparisons (p-value)	**RST**	**To Vehicle within -RST**	**To Vehicle within +RST**
Vehicle	<0.0001		
0.3 mg/kg CP55,940	0.0012	0.9991	>0.9999
1 mg/kg TCS-1102	0.0991	0.4998	>0.9999
0.3 mg/kg CP55,940 +1 mg/kg TCS-1102	0.1665	0.2961	>0.9999
3 mg/kg SR141716A	<0.0001	0.9996	>0.9999
40 mg/kg YNT-185	0.2945	0.9004	0.9977
3 mg/kg SR141716A + 40 mg/kg YNT-185	>0.9999	0.1216	0.2353

**Table S8.** Figure 2D ANOVA	**F(DFn,DFd)**
RST	F (1, 70) = 88.30
Treatment	F (6, 70) = 2.015
Interaction	F (6, 70) = 4.836

**Table S9.** Figure 3C Multiple Comparisons (p-value)	**RST**	**To Vehicle within -RST**	**To Vehicle within +RST**
Vehicle	>0.9999		
0.3 mg/kg CP55,940	0.9684	>0.9999	>0.9999
1 mg/kg TCS-1102	>0.9999	>0.9999	>0.9999
0.3 mg/kg CP55,940 +1 mg/kg TCS-1102	0.6972	>0.9999	0.9154
3 mg/kg SR141716A	0.9985	>0.9999	>0.9999
40 mg/kg YNT-185	>0.9999	0.9915	0.9999
3 mg/kg SR141716A + 40 mg/kg YNT-185	>0.9999	>0.9999	>0.9999

**Table S10.** Figure 3C ANOVA	**F(DFn,DFd)**
RST	F (1, 70) = 6.465
Treatment	F (6, 70) = 1.351
Interaction	F (6, 70) = 0.4251

**Table S11.** Figure 4A Multiple Comparisons (p-value)	**RST**	**To Vehicle within -RST**	**To Vehicle within +RST**
Vehicle	0.7095		
0.3 mg/kg CP55,940	>0.9999	>0.9999	0.9983
1 mg/kg TCS-1102	0.9999	>0.9999	>0.9999
0.3 mg/kg CP55,940 +1 mg/kg TCS-1102	>0.9999	>0.9999	0.9932
3 mg/kg SR141716A	0.9996	>0.9999	>0.9999
40 mg/kg YNT-185	>0.9999	>0.9999	0.9973
3 mg/kg SR141716A + 40 mg/kg YNT-185	0.9826	>0.9999	>0.9999

**Table S12.** Figure 4A ANOVA	**F(DFn,DFd)**
RST	F (1, 58) = 5.613
Treatment	F (6, 58) = 0.3170
Interaction	F (6, 58) = 0.3468

**Table S13.** Figure 5A Multiple Comparisons (p-value)	**RST**	**To Vehicle within -RST**	**To Vehicle within +RST**
Vehicle	0.9994		
3 mg/kg SR141716A	0.9931	>0.9999	0.9821
40 mg/kg YNT-185	0.3902	0.9613	0.9993
3 mg/kg SR141716A + 40 mg/kg YNT-185	0.9983	0.0051	<0.0001

**Table S14.** Figure 5A ANOVA	**F(DFn,DFd)**
RST	F (1, 72) = 0.4007
Treatment	F (3, 72) = 18.60
Interaction	F (3, 72) = 1.853

**Table S15.** Figure 5B Multiple Comparisons (p-value)	**RST**	**To Vehicle within -RST**	**To Vehicle within +RST**
Vehicle	0.0889		
3 mg/kg SR141716A	>0.9999	0.9998	0.3905
40 mg/kg YNT-185	0.9902	0.9301	0.9888
3 mg/kg SR141716A + 40 mg/kg YNT-185	0.9633	<0.0001	0.1677

**Table S16.** Figure 5B ANOVA	**F(DFn,DFd)**
RST	F (1, 72) = 2.211
Treatment	F (3, 72) = 24.05
Interaction	F (3, 72) = 2.692

**Table S17.** Figure 5C Multiple Comparisons (p-value)	**RST**	**To Vehicle within -RST**	**To Vehicle within +RST**
Vehicle	0.0147		
3 mg/kg SR141716A	>0.9999	0.9995	0.0014
40 mg/kg YNT-185	0.9980	>0.9999	0.1011
3 mg/kg SR141716A + 40 mg/kg YNT-185	>0.9999	0.0004	0.9433

**Table S18.** Figure 5C ANOVA	**F(DFn,DFd)**
RST	F (1, 72) = 4.110
Treatment	F (3, 72) = 21.22
Interaction	F (3, 72) = 2.999

**Table S19.** Figure 6A Multiple Comparisons (p-value)	**RST**	**To Vehicle within -RST**	**To Vehicle within +RST**
Vehicle	0.9964		
3 mg/kg SR141716A	0.9935	>0.9999	0.7679
40 mg/kg YNT-185	0.7813	>0.9999	0.9946
3 mg/kg SR141716A + 40 mg/kg YNT-185	0.1773	>0.9999	0.7541

**Table S20.** Figure 6A ANOVA	**F(DFn,DFd)**
RST	F (1, 72) = 4.122
Treatment	F (3, 72) = 1.611
Interaction	F (3, 72) = 2.027

**Table S21.** Figure 6B Multiple Comparisons (p-value)	**RST**	**To Vehicle within -RST**	**To Vehicle within +RST**
Vehicle	0.8555		
3 mg/kg SR141716A	>0.9999	0.9467	>0.9999
40 mg/kg YNT-185	0.0149	0.0687	0.5217
3 mg/kg SR141716A + 40 mg/kg YNT-185	0.8555	0.0269	0.9993

**Table S22.** Figure 6B ANOVA	**F(DFn,DFd)**
RST	F (1, 72) = 2.611
Treatment	F (3, 72) = 2.770
Interaction	F (3, 72) = 4.671

**Table S23.** Figure 6C Multiple Comparisons (p-value)	**RST**	**To Vehicle within -RST**	**To Vehicle within +RST**
Vehicle	0.9940		
3 mg/kg SR141716A	0.2636	0.9995	0.1615
40 mg/kg YNT-185	0.8681	0.4887	0.8525
3 mg/kg SR141716A + 40 mg/kg YNT-185	0.3994	0.9755	>0.9999

**Table S24.** Figure 6C ANOVA	**F(DFn,DFd)**
RST	F (1, 72) = 0.8982
Treatment	F (3, 72) = 2.353
Interaction	F (3, 72) = 3.951

**Table S25.** Figure 7A Multiple Comparisons (p-value)	**RST**	**To Vehicle within -RST**	**To Vehicle within +RST**
Vehicle	>0.9999		
3 mg/kg SR141716A	>0.9999	>0.9999	>0.9999
40 mg/kg YNT-185	0.9972	>0.9999	0.9979
3 mg/kg SR141716A + 40 mg/kg YNT-185	0.0100	0.3400	<0.0001

**Table S26.** Figure 7A ANOVA	**F(DFn,DFd)**
RST	F (1, 72) = 5.337
Treatment	F (3, 72) = 15.69
Interaction	F (3, 72) = 2.904

**Table S27.** Figure 7B Multiple Comparisons (p-value)	**RST**	**To Vehicle within -RST**	**To Vehicle within +RST**
Vehicle	0.9213		
3 mg/kg SR141716A	>0.9999	0.9997	0.9971
40 mg/kg YNT-185	0.9934	>0.9999	>0.9999
3 mg/kg SR141716A + 40 mg/kg YNT-185	>0.9999	0.0925	0.5904

**Table S28.** Figure 7B ANOVA	**F(DFn,DFd)**
RST	F (1, 72) = 1.273
Treatment	F (3, 72) = 5.53
Interaction	F (3, 72) = 0.2960

**Table S29.** Figure 7C Multiple Comparisons (p-value)	**RST**	**To Vehicle within -RST**	**To Vehicle within +RST**
Vehicle	0.9810		
3 mg/kg SR141716A	0.2829	0.9957	0.9974
40 mg/kg YNT-185	0.6569	>0.9999	0.9990
3 mg/kg SR141716A + 40 mg/kg YNT-185	0.1758	0.6323	0.0208

**Table S30.** Figure 7C ANOVA	**F(DFn,DFd)**
RST	F (1, 72) = 14.54
Treatment	F (3, 72) = 6.592
Interaction	F (3, 72) = 0.5422

**Table S31.** Figure 8A Multiple Comparisons (p-value)	**RST**	**To Vehicle within -RST**	**To Vehicle within +RST**
Vehicle	0.6977		
3 mg/kg SR141716A	0.9952	0.9988	0.0641
40 mg/kg YNT-185	0.5720	>0.9999	0.9998
3 mg/kg SR141716A + 40 mg/kg YNT-185	0.9943	>0.9999	0.2960

**Table S32.** Figure 8A ANOVA	**F(DFn,DFd)**
RST	F (1, 72) = 1.046
Treatment	F (3, 72) = 4.022
Interaction	F (3, 72) = 2.150

**Table S33.** Figure 8B Multiple Comparisons (p-value)	**RST**	**To Vehicle within -RST**	**To Vehicle within +RST**
Vehicle	0.9973		
3 mg/kg SR141716A	>0.9999	0.9217	0.9864
40 mg/kg YNT-185	0.9991	0.1613	0.8626
3 mg/kg SR141716A + 40 mg/kg YNT-185	0.9995	0.2111	0.8946

**Table S34.** Figure 8B ANOVA	**F(DFn,DFd)**
RST	F (1, 72) = 0.001529
Treatment	F (3, 72) = 3.467
Interaction	F (3, 72) = 0.3912

**Table S35.** Figure 8B Multiple Comparisons (p-value)	**RST**	**To Vehicle within -RST**	**To Vehicle within +RST**
Vehicle	>0.9999		
3 mg/kg SR141716A	0.5237	0.1183	0.0008
40 mg/kg YNT-185	0.5898	0.7578	0.0482
3 mg/kg SR141716A + 40 mg/kg YNT-185	0.9977	0.1951	0.7578

**Table S36.** Figure 8B ANOVA	**F(DFn,DFd)**
RST	F (1, 72) = 2.947
Treatment	F (3, 72) = 8.917
Interaction	F (3, 72) = 1.596
